# Can human-machine feedback in a smart learning environment enhance learners’ learning performance? A meta-analysis

**DOI:** 10.3389/fpsyg.2023.1288503

**Published:** 2024-01-10

**Authors:** Mengyi Liao, Kaige Zhu, Guangshuai Wang

**Affiliations:** ^1^School of Education, Pingdingshan University, Pingdingshan, Henan, China; ^2^National Engineering Research Center of Educational Big Data, Faculty of Artificial Intelligence in Education, Central China Normal University, Wuhan, Hubei, China

**Keywords:** feedback direction, feedback form, feedback technique type, human-machine feedback, meta-analysis, smart learning environment

## Abstract

**Objective:**

The human-machine feedback in a smart learning environment can influences learners’ learning styles, ability enhancement, and affective interactions. However, whether it has stability in improving learning performance and learning processes, the findings of many empirical studies are controversial. This study aimed to analyze the effect of human-machine feedback on learning performance and the potential boundary conditions that produce the effect in a smart learning environment.

**Methods:**

Web of Science, EBSCO, PsycINFO, and Science Direct were searched for publications from 2010 to 2022. We included randomized controlled trials with learning performance as outcome. The random effects model was used in the meta-analysis. The main effect tests and the heterogeneity tests were used to evaluate the effect of human-machine feedback mechanism on learning performance, and the boundary conditions of the effect were tested by moderating effects. Moreover, the validity of the meta-analysis was proved by publication bias test.

**Results:**

Out of 35 articles identified, 2,222 participants were included in this study. Human-machine interaction feedback had significant effects on learners’ learning process (*d* = 0.594, *k* = 26) and learning outcomes (*d* = 0.407, *k* = 42). Also, the positive effects of human-machine interaction feedback were regulated by the direction of feedback, the form of feedback, and the type of feedback technique.

**Conclusion:**

To enhance learning performance through human-machine interactive feedback, we should focus on using two-way and multi-subject feedback. The technology that can provide emotional feedback and feedback loops should be used as a priority. Also, pay attention to the feedback process and mechanism, avoid increasing students’ dependence on machines, and strengthen learners’ subjectivity from feedback mechanism.

## 1 Introduction

The application of new technologies such as cloud computing, big data and artificial intelligence have prompted a revolutionary change in education. As a new form, smart learning environment integrates relevant technologies and devices to provide personalized learning content and real learning experience through various human-machine interaction (Hew and Kadir, 2016). In human-machine feedback, machines collect and analyze learning data to provide learners with personalized feedback to improve their learning performance. However, not all human-machine feedback can achieve the desired effect, and feedback can be effective only when learners understand the feedback and are willing to act on it (Price et al., 2010). For example, the generative artificial intelligence such as ChatGPT adopts the technology of Reinforcement Learning from Human Feedback (RL-HF), which has the ability to improve output according to user’s feedback. It could continuously self-iteration based on user’s feedback (Ouyang et al., 2022; Shen et al., 2023), which is conducive to the formation of continuous feedback and feedback loops. However, the educational application of ChatGPT is still in the stage of exploration, as well as its feedback characteristics and influence on learners’ learning performance are still unknown. Therefore, whether the feedback direction, feedback form, and feedback technology type has effect on learners’ learning performance? and what is the boundary condition of the effect? Exploring these problems is of great significance to the current application of smart learning environment.

The human-machine interactive feedback has certain directivity, such as one-way feedback dominated by computer, two-way feedback with two subjects (computer and learner) (Dong, 2020), and multi-subject feedback (computer, learner, peer and teacher). One-way feedback is dominated by computer, which is easy to ignore learners’ initiative, and gradually make learners lose their learning status. For example, the automatic planning of learning paths by computers simplifies the learning process of learners’ self-reflection and self-regulation, which to some extent affects the quality of education (Zhang and Liang, 2020). At the same time, some human-machine interactions that incorporate irrelevant factors may obscure the learning focus and increase the cognitive load of learners, decreasing the effectiveness of learning (Zhang, 2018). Two-way feedback can give play to the advantages of the computer, and highlight the learner’s subjectivity, which is of great value for improving the learner’s subjective, cultivating higher-order ability and strengthening emotional interaction (Baker, 2016). For example, using incentive-based online dialogue agents, learners with low participation are motivated to change their behaviors by expressing common emotions (Xie et al., 2021). Programming training supported by tools such as ChatGPT can effectively improve students’ programming skills through human-machine collaborative coding and collaborative debugging (Chen et al., 2023), but which ignores feedback from peers and teachers. Multi-subject feedback integrates the advantages of computers, learners, peers, teachers and other multi-agents to improve learners’ learning performance. For example, anthropomorphic robots that integrate the advantages of multiple agents, can reduce learners’ anxiety level and significantly improve learners’ foreign language learning performance, learning satisfaction and learning motivation (Hong et al., 2016). It has great value to improve the effect of human-machine feedback by analyzing human-machine feedback directions and its influence on learners’ learning performance of existing studies.

The feedback form in smart learning environment include static feedback and dynamic feedback (Dong et al., 2021). Among them, static feedback means that the preset learning resources flow from computer to learner, and the learning content is difficult to adapt to the change of the learner’s learning state, and cannot meet the learner’s learning needs in real time. For example, by providing preset learning content to learners, virtual reality-based smart learning environment has a positive impact on learners’ learning interest and motivation, but has no significant impact on their academic performance (Parong and Mayer, 2018). Dynamic feedback can collect the learners’ learning state in real time, and accurately adjust the learning content, which is in line with the dynamic changes in the learning process. For example, the intelligent voice tutoring system can perceives learners’ oral performance and adjusts the strategies to improve learners’ oral ability (Mohammadzadeh and Sarkhosh, 2018). However, the boundary conditions for the effectiveness of different feedback forms are still unclear. It is significance to analyze the influence of different feedback forms on learners’ learning performance and explore the best feedback form.

The smart learning environment contains many feedback techniques which have different effects on learners’ learning performance. The feedback will be affected by the context in which learners are more likely to perceive feedback information and increase learning engagement (Noble et al., 2020). For example, by providing different scenes to assist English vocabulary learning, social robots can reduce learners’ anxiety and enhance learners’ pleasure, motivation and attitude (Alemi et al., 2015). At the same time, the combination of technology and education promotes the application of VR in K-12 science course (Georgiou et al., 2021), and the virtual laboratory can improve students’ practical ability in physics, chemistry, geography, biology (Sanfilippo et al., 2022). Force feedback technology has also been applied to physical experiments to enrich students’ experience (Magana et al., 2019). In addition, the intelligent writing evaluation system can improve learners’ self-efficacy and thus improve their performance (Wilson and Roscoe, 2019). Adaptive mathematics learning system can automatically adjust the teaching order or provide process-oriented feedback according to the error rate after the completion of each task (Bush, 2021). Artificial intelligence technology represented by ChatGPT can improve learners’ computational thinking skills, programming self-efficacy and motivation (Ramazan and Fatma, 2023b). With the advancement of technology and the research of human-machine feedback, there will be more and more human-machine feedback technologies applied in teaching. It is helpful to improve the effectiveness of human-machine feedback by analyzing the influence of different feedback technologies.

**FIGURE 1:**
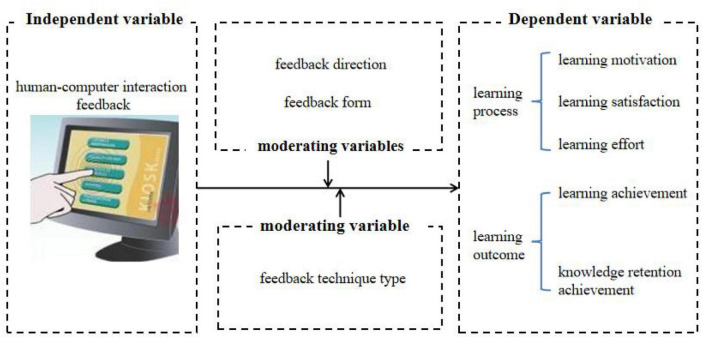
Structural relationship between variables.

In summary, many researchers have conducted experimental and quasi-experimental studies on learners’ learning process and learning outcome under different feedback directions, feedback forms, and feedback technology types. The learning process emphasizes individual experience, accompanied by different emotions and wills. It includes learning motivation, learning satisfaction and learning effort. The learning outcome is the intrinsic and lasting change in learners’ knowledge and skills through learning activities, including learning achievement and knowledge retention achievement. However, whether the human-machine feedback mechanism in smart learning environment is stabilized in improving learners’ learning performance is still controversial. Therefore, in order to explore the effect of human-machine feedback mechanism on learning performance and its potential boundary conditions, meta-analysis method is adopted. In this study, human-machine interaction feedback was the independent variable, and learning performance was the dependent variable including the learning process and learning outcome. Also, factors such as feedback direction, feedback form, and feedback technique type were considered as moderating variables. The structural relationship between the variables studied in this paper is shown in Figure 1.

We focus on the following research questions:

(1) Does human-machine feedback mechanism in smart learning environments enhance students’ learning performance? How effective are the different human-machine feedback mechanisms?
(2) What are the potential boundary conditions for the effects of human-machine feedback? Do the direction, form, and technology type of human-machine feedback play a moderating role in learners’ learning process and learning outcome?

## 2 Materials and methods

The study was designed in accordance with the PRISMA Statement for Reporting Systematic Reviews and Meta-analysis of Studies (Moher et al., 2009).

### 2.1 Search and screening

Literature for this meta-analysis was conducted by searching the online databases of Web of Science, EBSCO, PsycINFO, and Science Direct, using keywords such as “intelligent tutoring systems,” “intelligent interactive education,” “intelligent learning environment,” “online feedback teaching,” “intelligent learning companion,” and “virtual teacher.” The literature span was set from 2010 to 2022.

Using these specified terms, we identified 726 articles. Through backtracking, additional 6 articles were identified. First, titles and abstracts of the 732 articles were screened to determine their relevance to the study. This resulted in the exclusion of 515 articles (383 articles were duplicate; 7 articles were conference abstracts; and 125 articles were not experimental or quasi-experimental study). Thus 217 articles remained.

**FIGURE 2:**
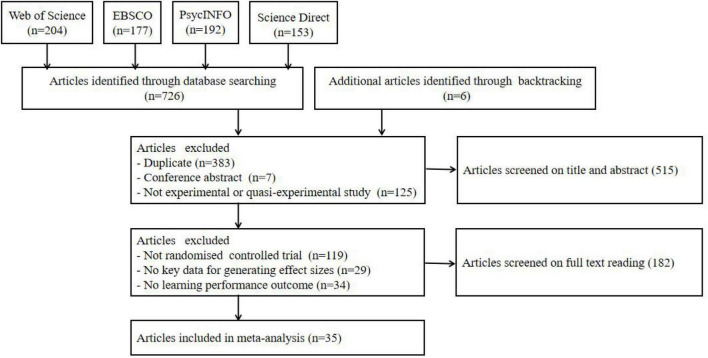
Flow chart of the search strategy of the study.

Second, of the 217 articles that were identified, the title, abstract and method section of each record were systematically reviewed and considered for inclusion. The inclusion and exclusion criteria for the literature were as follows: (1) Include only experimental and quasi-experimental studies related to human-machine interaction feedback in smart learning environment; (2) Include comparison studies with and without human-machine interaction feedback, and exclude studies without a control group; (3) Key data for generating effect sizes, such as sample size, mean, standard deviation, etc., were reported in the study and otherwise excluded; (4) At least one of the five dependent variables was reported in the study, otherwise they were excluded. Three independent reviewers rated the articles (one postgraduate, one *post-doc* and a professor). Agreement between raters was between 81 and 94%. Differences between reviewers were resolved by consensus. This resulted in the exclusion of 182 articles (119 articles have no randomized controlled trial; 29 articles have no key data for generating effect sizes; and 34 articles have no learning performance outcome). Thus 35 articles were included in the meta-analysis. According to statistical theory, the results of meta-analysis will be accurate and reliable when the sample size is not less than 30, and the results will be more desirable if the sample size is more than 50. Therefore, the sample size of this study meets the basic requirements of meta-analysis (Tipton, 2014). The flow chart of selected articles were demonstrates in Figure 2.

### 2.2 Coding of studies

The main coding elements in this study were as follows: feedback direction (one-way/two-way/multi-subject), feedback form (static/dynamic), and feedback technology type (virtual reality/educational robot/intelligent tutor system/intelligent classroom/intelligent interactive learning system). Coding was done by the first author and the corresponding author. Overall agreement between authors was 97%. The authors reached an agreement and the differences were resolved thoroughly through comprehensive discussion. The results of literature coding are shown in Table 1. If the included literature did not provide effect sizes directly, effect sizes could be counted by sample size, mean, standard deviation, and other data. Multiple experiments in the same literature could be split into multiple effect sizes if the experimental variables were moderating variables, otherwise they were combined into the same effect size. Accordingly, 11 independent effect sizes were generated for learning motivation, 5 for learning satisfaction, 10 for learning effort, 35 for learning achievement, and 7 for knowledge retention achievement. Ultimately, 68 independent effect sizes from 35 articles were included in the meta-analysis.

**TABLE 1:** The results of literature coding.

References	Experimental sample size & control sample size	Feedback direction	Feedback form	Feedback technique type	Learning performance
Liu et al., 2022	170 & 192	Two-way	Dynamic	Virtual reality	Learning process
Chung and Lin, 2022	112 & 103	Two-way	Dynamic	Virtual reality	Learning outcome
Danial et al., 2021	36 & 42	Two-way	Dynamic	Virtual reality	Learning outcome
Thomas, 2021	215 & 211	Two-way	Dynamic	Blended learning	Learning process
Hwang et al., 2020	43 & 40	Two-way	Dynamic	Virtual reality	Learning outcome
Higinio et al., 2020	78 & 65	Multi-subject	Dynamic	Intelligent tutor system	Learning outcome
Kathryn et al., 2018	116 & 118	Two-way	Dynamic	Intelligent tutor system	Learning outcome
Ahmad and Mehdi, 2018	15 & 15	Two-way	Dynamic	Intelligent tutor system	Learning outcome
Benjamin et al., 2018	28 & 48	Two-way	Dynamic	Intelligent tutor system	Learning outcome
Ji et al., 2018	1087 & 721	Two-way	Static	Intelligent tutor system	Learning outcome
Guo, 2017	31 & 29	Two-way	Static	Virtual reality	Learning outcome
Qiao, 2017	42 & 41	One-way	Static	Educational robots	Learning process
Long and Aleven (2017)	56 & 56	Two-way	Dynamic	Intelligent tutor system	Learning outcome
Octavio et al., 2017	33 & 27	One-way	Static	Educational robots	Learning process
Hong et al., 2016	25 & 27	Multi-subject	Static	Educational robots	Learning outcome
Seong-won and Youngjun, 2016	14 & 26	One-way	Static	Educational robots	Learning process
Peng, 2016	30 & 30	Two-way	Static	Virtual reality	Learning outcome
Michael and Fox, 2015	71 & 71	One-way	Static	Virtual reality	Learning process
Alemi et al., 2015	30 & 16	Two-way	Static	Educational robots	Learning process
Carme and Juan, 2015	9 & 12	One-way	Static	Educational robots	Learning outcome
Kosta and Boris, 2015	59 & 58	Two-way	Dynamic	Intelligent tutor system	Learning outcome
Hsiao et al., 2015	30 & 27	Two-way	static	educational robots	learning outcome
Gwen et al., 2014	147 & 141	One-way	Static	Educational robots	Learning process
Zafar and Albidewi, 2015	29 & 28	Two-way	Dynamic	Intelligent tutor system	Learning outcome
Park, 2014	61 & 52	One-way	Static	Educational robots	Learning process
Chen et al., 2013	30 & 30	Multi-subject	Static	Educational robots	Learning outcome
Mostow et al., 2013	88 & 90	Two-way	Dynamic	Intelligent tutor system	Learning process
Ivon et al., 2013	41 & 41	Two-way	Dynamic	Intelligent tutor system	Learning outcome
Wijekumar et al., 2012	64 & 66	Two-way	Dynamic	Intelligent tutor system	learning outcome
Bettina and Jawaharlal, 2012	174 & 86	Two-way	Static	Educational robots	Learning process
Steve, 2012	38 & 37	Multi-subject	Static	Educational robots	Learning outcome
Park, 2012	34 & 28	One-way	Static	Educational robots	Learning process
D’Mello et al., 2012	24 & 24	Two-way	Dynamic	Intelligent tutor system	Learning outcome
Park and Kim, 2011	28 & 29	One-way	Static	Educational robots	Learning process
Carole et al., 2010	34 & 39	Two-way	Static	Intelligent tutor system	Learning outcome

The learning process involves learning motivation, learning satisfaction and learning effort. The learning outcome includes learning achievement and knowledge retention achievement.

### 2.3 Statistical analysis

We used the Comprehensive Meta-Analysis (CMA version 3.0) software to conduct meta-analysis. The statistic analysis process was as follows: (1) Analyze the data with a random effects model; (2) Conduct main effect tests and the heterogeneity tests to evaluate the effect of human-machine feedback mechanism on learning performance; (3) Use moderating effects model to test the boundary conditions of the effect; (4) Prove the validity of the meta-analysis by publication bias test.

## 3 Results

### 3.1 Main effects

In this study, Comprehensive Meta-Analysis version 3.0 (CMA 3.0) was used to conduct meta-analysis, and a random effects model was used for main effects testing. Hedges’ g was selected as the effect size, and Cohen’s d was used to estimate bias. The study analyzed the main effects of learning process and learning outcome, and the results were shown in Table 2. According to the interpretation of effect sizes in studies related to education, 0.2, 0.5, and 0.8 are seen as the boundaries of small, medium, and large effect sizes, and 1.2 and 2.0 are seen as the boundaries of great and huge effects (Van et al., 2015). As seen in Table 2, the random effects model showed that human-machine interaction feedback has an overall significant positive effect on learning process & outcome (effect size = 0.457, 95% CI [0.348, 0.576], *p* < 0.001). Therefore, the results of the effect size show that, on the whole, human-machine interaction feedback has a middle level positive effect on learning process & outcome.

**TABLE 2:** Main effects and heterogeneity test.

Dependent variable	*k*	*d*	95% CI	Heterogeneity			Tau*-*squared			
				*Q*	*p*	*I* ^2^	Tau*-*Squ	SE	Variance	Tau
Learning process	26	0.594	[0.294, 0.894]	404.986	<0.001	93.827	0.546	0.192	0.037	0.739
Learning outcome	42	0.407	[0.304, 0.511]	256.547	<0.001	84.019	0.070	0.033	0.001	0.264
Learning Process & Learning outcome	68	0.457	[0.348, 0.576]	675.383	<0.001	90.080	0.157	0.059	0.004	0.397

k denotes the number of effect sizes, N denotes the sample size, d denotes the effect size, and CI denotes confidence interval.

As shown in Table 2, human-machine interaction feedback has a significant positive effect on learning process (effect size = 0.594, 95% CI [0.294, 0.894], *p* < 0.001), indicating that human-machine interaction feedback has a moderately high level positive effect on learning process. In addition, human-machine interaction feedback also has a significant positive effect on learning outcome (effect size = 0.407, 95% CI [0.304, 0.511], *p* < 0.001), indicating that human-machine interaction feedback has a moderately low level positive effect on learning outcome. In summary, human-machine interaction feedback can enhance learners’ learning process and learning outcomes to a certain extent.

### 3.2 Heterogeneity test

Table 2 presents the heterogeneity test results for human-machine interaction feedback on the learning process and learning outcomes. The *Q* tests were all significant (*p* < 0.001), indicating significant heterogeneity in the effect sizes of the dependent variable. *I*^2^ revealed that the learning process and learning outcome accounted for 90.080% of the total variance, and according to the criteria for determining the degree of heterogeneity of meta-analytic effect sizes proposed by Higgins et al. (2003), the total variance of the learning process and learning outcome was high in this study. Among them, the proportion of variance in learning process and learning outcome caused by real differences in effect sizes was 93.827 and 84.019% of the total variance, respectively, both with high degrees of heterogeneity, indicating that there may be potential moderating variables for the effect of human-machine interaction feedback (Xie et al., 2016). It is necessary for the study to conduct a moderating effect test for each dependent variable and then examine the feedback effect’s boundary conditions.

### 3.3 Moderating effects

The test of the moderating effect of human-machine interaction feedback on learning achievement were shown in Table 3. In learning achievement, feedback direction played a significant moderating role (*Q* = 6.097, *p* < 0.05), in which the promotion effect of two-way feedback and multi-subject feedback on learning achievement was significantly greater than that of one-way feedback; meanwhile, the moderating effect of feedback type of technique also played a significant moderating effect on learning achievement (*Q* = 7.314, *p* < 0.05). It was worth noting that the moderating effect of feedback form was not significant (*p* > 0.05), but the statistical results showed that both static feedback and dynamic feedback can significantly promote learning achievement.

**TABLE 3:** Test of the moderating effect of human-machine interaction feedback on learning achievement.

Moderating variable		*k*	*d*	95% CI	Heterogeneity		
					*Q*	df	*p*
Feedback direction	One-way feedback	2	0.036	[−0.350, 0.421]	6.097	2	0.047
	Two-way feedback	30	0.441**	[0.321, 0.560]			
	Multi-subject feedback	3	0.646**	[0.351, 0.940]			
Feedback form	Static feedback	19	0.454*	[0.317, 0.590]	0.011	1	0.916
	Dynamic feedback	16	0.439*	[0.217, 0.662]			
Feedback technique type	Educational robots	8	0.667**	[0.372, 0.961]	7.314	2	0.026
	Intelligent tutor system	25	0.311**	[0.206, 0.416]			
	Other technology types	2	1.358*	[0.074, 2.643]			

95% CI refers to the 95% confidence interval of the effect size, and Q represents the Q test for between-group heterogeneity, **p < 0.01, *p < 0.05.

In terms of knowledge retention (see Table 4), the moderating effects of feedback direction, feedback form and the type of feedback technology were all not significant (*p* > 0.05).

**TABLE 4:** Test of the moderating effect of human-machine interaction feedback on knowledge retention.

Moderating variable		*k*	*d*	95% CI	Heterogeneity		
					*Q*	df	*p*
Feedback direction	One-way feedback	1	0.547	[−0.333, 1.427]	0.384	1	0.536
	Two-way feedback	6	0.251	[−0.074, 0.576]			
Feedback form	Static feedback	5	0.351	[−0.053, 0.755]	0.747	1	0.387
	Dynamic feedback	2	0.147	[−0.077, 0.372]			
Feedback technique type	Educational robots	1	0.547	[−0.333, 1.427]	0.254	1	0.447
	Intelligent tutor system	5	0.028	[−0.236, 0.293]			

In terms of learning motivation, the amount of independent effect of feedback form on learning motivation was insufficient, so no moderating effect test was done. As shown in Table 5, the moderating effect of feedback direction was not significant (*p* > 0.05), but the statistical results showed that both one-way feedback and multi-subject feedback can significantly promote learning motivation. Meanwhile, the type of feedback technology significantly moderated learners’ motivation (*Q* = 22.128, *p* < 0.05), where the educational robot was significantly more effective in promoting learning motivation than other technology types.

**TABLE 5:** Test of the moderating effect of human-machine interaction feedback on learning motivation.

Moderating variable		*k*	*d*	95% CI	Heterogeneity		
					*Q*	df	*p*
Feedback direction	One-way feedback	10	1.103**	[0.370, 1.836]	0.977	1	0.323
	Multi-subject feedback	1	1.588*	[0.965, 2.212]			
Feedback technique type	Educational robots	8	1.773**	[0.936, 2.609]	22.128	1	0.000
	Other technology types	3	−0.377*	[−0.697, −0.056]			

**p < 0.01, *p < 0.05.

The independent effect size of the feedback form on learning satisfaction was insufficient, so no moderating effect test was done. Table 6 shows that feedback direction significantly moderates learners’ learning satisfaction (*Q* = 29.786, *p* < 0.05), where multi-subject feedback has a significantly greater facilitation effect on learning satisfaction than one-way feedback and two-way feedback; also, feedback technology type significantly moderates learners’ learning satisfaction (*Q* = 16.594, *p* < 0.05), where educational robots have a significantly greater facilitation effect on learning.

**TABLE 6:** Test of moderating effect of human-machine interaction feedback on learning satisfaction.

Moderating variable		*k*	*d*	95% CI	Heterogeneity		
					*Q*	df	*p*
Feedback direction	One-way feedback	3	−0.510*	[−0.934, −0.085]	29.786	2	0.000
	Two-way feedback	1	0.778*	[0.151, 1.406]			
	Multi-subject feedback	1	1.450**	[0.839, 2.061]			
Feedback technique type	Educational robots	2	1.118**	[0.460, 1.776]	16.594	1	0.000
	Other technology types	3	−0.510*	[−0.934, −0.085]			

**p < 0.01, *p < 0.05.

As shown in Table 7, feedback direction, feedback form, and feedback technique type showed significant moderating effects on learning effort (*p* < 0.05). Among them, the facilitation effect of multi-subject feedback on learning effort was significantly greater than that of one-way and two-way feedback; the facilitation effect of static feedback on learning effort was significantly greater than that of dynamic feedback; and the facilitation effect of educational robots on learning effort was significantly greater than that of other technology types.

**TABLE 7:** Test of the moderating effect of human-machine interaction feedback on learning effort.

Moderating variable		*k*	*d*	95% CI	Heterogeneity		
					*Q*	df	*p*
Feedback direction	One-way feedback	5	0.300	[−0.168, 0.768]	20.317	2	0.000
	Two-way feedback	4	0.288	[−0.034, 0.609]			
	Multi-subject feedback	1	1.922**	[1.264, 2.580]			
Feedback form	Static feedback	9	0.480**	[0.160, 0.800]	8.536	1	0.003
	Dynamic feedback	1	−0.168	[−0.462, 0.126]			
Feedback technique type	Educational robots	6	0.757**	[0.364, 1.151]	15.268	2	0.000
	Intelligent tutor system	1	−0.168	[−0.462, 0.126]			
	Other technology types	3	−0.058	[−0.302, 0.186]			

**p < 0.01.

### 3.4 Publication bias test

This study performed publication bias tests by fail-safe number and Egger linear regression. As shown in Table 8, the fail-safe number was greater than 5*k* + 10 (*k* refers to the number of independent effect sizes included in meta-analysis) for learning motivation, learning satisfaction, learning effort, learning achievement, and knowledge retention achievement, indicating a low likelihood of publication bias on the five dependent variables. Using the Egger linear regression analysis method, *p*-values were greater than 0.05 for learning satisfaction, learning effort, and knowledge retention achievement, indicating a low likelihood of publication bias; *p*-values were less than 0.05 for learning motivation and learning achievement, indicating a possible publication bias. The fail-safe number indicates that there is a low possibility of publication bias in this study, while the Egger linear regression indicate a possible publication bias in learning motivation and learning effort. In view of this contradiction, the trim-and-fill analyses was used and found that the overall effect size was still positive and significant after correction (*d* = 0.461, 95% CI [0.208, 0.713], *p* < 0.001). There was no significant change in the effect size after trim-and-fill, so it can be considered that the results of this meta-analysis were less affected by publication bias and there was no significant publication bias.

**TABLE 8:** Publication bias test.

Dependent variable	Rosenthal’s N_fs_	Egger’s intercept	*SE*	95% CI	*P*
Learning motivation	232	11.366	2.390	[5.958, 16.773]	0.001
Learning satisfaction	44	10.820	3.757	[−1.136, 22.777]	0.063
Learning effort	111	6.264	3.459	[−1.712, 14.241]	0.108
Learning achievement	1900	1.534	0.541	[0.434, 2.634]	0.007
Knowledge retention achievement	67	−0.049	2.324	[−6.025, 5.927]	0.984

## 4 Discussion

This is the first known meta-analysis to investigate the effects of human-machine feedback from three aspects: feedback direction, feedback form, and feedback technique type. In similar studies, some focus on the feedback effect of a technology, some analyze the feedback strategy of a subject, and some compare different feedback forms, but comprehensive analysis of moderating effects has not carried out yet. A meta-analysis of computer programming education indicated that the effect sizes differed only marginally between the instructional approaches and conditions - however, metacognition-based feedback teaching and visual feedback teaching were especially effective (Scherer et al., 2020). Regrettably, the subject and teaching strategies in this study limit the generalization of the conclusions. The feedback effect is affected by the feedback technique type. Augmented reality (AR) provides learners with immersive situational feedback. A study on the effect of language learning using AR technology found that AR has a large effect on learners’ language gains and a medium effect on learners’ motivation. A systematic review of online peer feedback tools found that the effect sizes seemed to vary widely across studies, indicating that implementation details are important, but the factors that influence implementation details and effects are still not fully presented in this study (Zong et al., 2021).

This research was carried out by the meta-analysis to determine the effectiveness and its potential boundary conditions of human-machine feedback. The result showed that human-machine feedback had significant effects on learners’ learning process (*d* = 0.594, *k* = 26) and learning outcomes (*d* = 0.407, *k* = 42), and the positive effects of human-machine feedback were moderated by the feedback direction, the feedback form and the feedback technique type. The findings obtained from the research are discussed below.

### 4.1 The first sub-problem of the study: the effect of human-machine feedback on learning performance

In the first sub-problem of the study, it was examined whether the use of human-machine feedback made a significant effect on learners’ learning process. The main effects test of the meta-analysis found that adding feedback to human-machine interaction could increase learning motivation, learning satisfaction, and learning effort compared to no-feedback. Human-machine interaction feedback enhanced learners’ subjectivity and promoted affective interaction, as evidenced by increased learning motivation, learning satisfaction, and learning effort, consistent with the findings of numerous previous empirical studies (Hattie, 2012; Baker, 2016; Yorganci, 2022). First, technology-enabled human-machine interaction helped present authentic and effective feedback-based learning tools and learning scenarios, making the learning process intuitive, efficient, and interesting, which was conducive to enhancing learning satisfaction (Yang and Ren, 2019; Fatma and Ramazan, 2022). A technology-supported learning environment and related learning tools facilitated the visual presentation of learning content and learning tasks, and students could gain an immersive learning experience; timely information feedback during the learning process provided scaffolding for students to construct knowledge. The availability of relevant technology tools further facilitated the active construction of knowledge by learners. Second, human-machine interaction feedback stimulated endogenous motivation for learning, transforms students’ inherent learning concepts and habits, and helped improve learning efforts. In the process of human-machine interaction, when the machine senses learners’ negative emotional or behavioral, it will help students adjust their emotional attitudes and restrain bad behaviors through timely feedback, so as to improve learners’ learning efforts (Handley and Williams, 2011). Third, human-machine interactive feedback can adjust students’ motivation psychology in real time and improve learners’ learning motivation. Human-machine interaction feedback helped students clarify what they knew, what they were doing, how far they had progressed, how they could adjust to improve their current learning situation. Through the above ways, expectation motivation can be improved and anxiety motivation can be reduced. Feedback can also help students clarify how to further approach the learning goal, strengthen their desire for knowledge, and achieve the purpose of improving learning motivation (Bellon et al., 2020).

On the other hand, the first sub-problem also examined whether the use of human-machine feedback had a significant effect on learners’ learning outcomes. The main effects test of the meta-analysis showed that adding feedback to human-machine interaction could enhance learners’ learning achievement and knowledge retention effects, consistent with the findings of many previous empirical studies (VanLehn, 2011; Steenbergen-Hu and Cooper, 2014; Kulik and Fletcher, 2016). First, human-machine interaction feedback might create an adaptive learning environment for students, which could help improve learning achievement and knowledge retention (Wang and Chen, 2022). Human-machine feedback focused on difficult knowledge to help students with highly constrained tasks. Also, it can help to externalize internal thinking, decompose complex cognitive processes, and achieve knowledge retention. At the same time, it can created knowledge to enhance learning achievement through the absorption and integration of feedback content. Second, human-machine interaction in the smart learning environment broke through traditional learning methods and transformed learning from repetitive memory or practice to higher-order thinking and deep learning, extending learners’ learning capabilities and thus improving their learning achievement. Through human-machine interaction feedback, the machine could sense the learner’s learning status and learning style and feedback personalized learning resources and learning paths to the learner accordingly, during which the learner could repeatedly communicate and replay the learning trajectory for deep reflection and optimization to achieve optimal knowledge construction results (Ramazan et al., 2022). Based on the above two points, it can be said that the use of human-machine interaction feedback is effective in improving learners’ learning outcomes.

### 4.2 The second sub-problem of the study: the boundary conditions of the human-machine feedback effect

In the second sub-problem of the study, the boundary conditions of the human-machine feedback effect were examined. From the results of the moderating effects, there were certain boundary conditions for the effects of human-machine interaction feedback on learning achievement, in which feedback direction and feedback technique type had significant moderating effects, while the moderating effects of feedback form was not significant. In terms of learning motivation, only the type of feedback technique had a significant moderating effect, while the moderating effects of the other moderating variables were not significant. On learning satisfaction, feedback direction and type of feedback technique had a significant moderating effect on learning satisfaction. On learning effort, feedback direction, feedback form, and feedback technique type all showed significant moderating effects.

Regarding learning achievement, the direction of feedback and the type of feedback technique had a significant moderating effect on it. The facilitation effect of two-way feedback and multi-subject feedback were significantly greater than that of one-way feedback, as the findings of Agapito and Rodrigo (2018). First, the current one-way feedback was mainly machine to student, compared with two-way feedback and multi-subject feedback, which provided feedback loops for students, promoted deep learning and higher-order thinking of learners, and were conducive to the improvement of learners’ learning performance. Second, multi-subject feedback expanded the interaction subject and sources of feedback information, enriched students’ perception of the learning environment, and students perceive and understand the learning process through a variety of sensing devices, which promoted the embodiment of students’ knowledge understanding and contributed to the improvement of learners’ performance (Wang and Zheng, 2015). Among the different types of feedback technologies, educational robots and intelligent tutor systems contributed significantly more to learning performance than the other technology types, as the findings of Kulik and Fletcher (2016). First, compared with feedback technology types such as virtual reality, educational robots and intelligent tutor systems focused more on the smart learning environment which helped to provide personalized learning paths for students, thereby enhancing their learning achievement. Second, with the development of emerging technologies such as the internet of things and bionic technology, educational robots were increasingly highlighting their advantages in human-machine interaction, which enabled students to perceive knowledge, unify cognition, manage emotions and regulate learning motivation through human-machine interaction, enhancing learners’ subjectivity and higher-order learning abilities (Casad and Jawaharlal, 2012). Therefore, in applying human-machine interaction for wisdom learning, we should expand the direction of human-machine interaction feedback and information sources, focus on two-way and multi-subject feedback, and promote the formation of feedback loops in the learning process. At the same time, priority was given to the use of technology forms such as educational robots and intelligent tutors to carry out personalized teaching through adaptive technology, enhance the subjectivity of learners’ learning through human-machine interaction, design adaptive teaching resources and teaching models, and ultimately improve students’ learning achievement.

In terms of learning motivation, the feedback technology types had a significant moderating effect on it. Educational robots had a greater facilitation effect than other technology types. Educational robots were more likely to promote learner motivation. This result was consistent with the findings of Kim and Kang (2010). Technologies such as artificial intelligence, language recognition, and bionic technology are becoming increasingly mature, greatly enhancing the authenticity of educational robots’ interactions with learners and improving learners’ sense of social presence, thereby enhancing learners’ motivation to learn (Ramazan and Fatma, 2023a). Social presence is the extent to which the learner is perceived as a real person during human-machine interaction and the extent to which people perceive a connection with other people (Biocca et al., 2003). In this scenario, the learner demonstrates his emotional and interpersonal skills as a real person, and the learner’s spiritual and intellectual engagement can greatly enhance the learner’s motivation to learn. Therefore, the design of educational robot hardware and supporting resources should be strengthened to enhance learners’ learning experience, while the mechanism of educational robots on learning motivation should be explored through multidisciplinary collaborative research in brain science, psychology, and education. The application and practice model of educational robots should be innovated to further enhance the role of educational robots in promoting learners’ learning motivation.

In terms of learning satisfaction, the direction of feedback and the type of feedback technique had a significant moderating effect on it. Regarding the direction of feedback, the facilitation effect of multi-subject feedback was significantly greater than that of two-way and one-way feedback. This result was consistent with the findings of Dong et al. (2021). Multi-subject feedback integrated the interaction between teachers, peers, individual learners and machines, which was more suitable for practical teaching scenarios and more conducive to the implementation of emotional feedback, helping learners to improve learning satisfaction (Dong et al., 2021). One-way feedback and two-way feedback blurred the learning subject and changed learners from full participation to partial participation, which affected learners’ learning satisfaction to a certain extent. Among the different types of feedback technologies, educational robots contributed significantly more to learning satisfaction than other technology types, and this result was consistent with the findings of Zhou et al. (2019), where educational robots were more likely to integrate with new technologies such as augmented reality, virtual reality, 3D printing, bionic technology, and voice interaction to enhance learners’ interest in learning and learning satisfaction. Therefore, in human-machine interaction feedback, we should pay attention to the information feedback of relevant interaction subjects, strengthen the emotional feedback between different interaction subjects, mobilize emotional factors to carry out active learning, strengthen beliefs, and enhance learners’ learning satisfaction.

In terms of learning effort, feedback direction, feedback form, and type of feedback technique all had significant moderating effects on it. Among them, the facilitation effect of multi-subject feedback was significantly greater than that of one-way and two-way feedback. The facilitation effect of educational robots was significantly greater than that of other technology types, similar to the findings of learning satisfaction. Multi-subject feedback and educational robotics enhanced learners’ learning satisfaction by providing them with various emotional support, promoting a trusting feedback environment among learning subjects, making them more receptive to external feedback, and motivating them to respond positively to external feedback and actively engage in learning. However, in terms of feedback form, static feedback was significantly more effective in promoting learning effort than dynamic feedback. Dynamic feedback relied on the adaptive technology of artificial intelligence, which obtained learners’ behavioral, emotional, and cognitive states in real-time and provided students with feedback on precise learning resources and learning paths, and learners’ learning behaviors were manipulated by the intelligent learning environment, resulting in learners’ over-reliance on machines, passive acceptance of feedback information in the learning process, and lack of cognitive integration of feedback information, which affected learners’ learning effort to some extent (Baker, 2016; Wang and Chen, 2022). Therefore, in the process of human-machine interaction feedback, it was important to strengthen the attention to the feedback process and mechanism, not to aggravate students’ dependence on the machine by emphasizing human-machine interaction feedback, to strengthen learners’ subjectivity from the level of feedback mechanism, and to support learners’ cognitive integration of feedback information, and then to actively regulate motivation, take action, and enhance learning effort.

## 5 Conclusion

This study used meta-analysis to explore the effect of human-machine interaction feedback and its potential boundary conditions. The results of the study were as follows. In this study, the effect of human-machine feedback on learning performance in smart learning environment was examined. The research was carried out according to the experimental design of meta-analysis. As a result of the research, human-machine interaction feedback in smart learning environment has a significant effect on learners’ learning process, as evidenced by the increase in learners’ motivation, learning satisfaction, and learning effort. Meanwhile, the effect of human-machine interaction feedback on learning outcomes was significant, mainly in the form of improved learning achievement and knowledge retention. Also, the positive effect of human-machine interaction feedback was moderated by factors such as feedback direction, feedback form, and feedback technique type. Therefore, only when the feedback mechanism is carefully used in human-machine interaction, can the positive learning effect be generated. Specifically, focus on two-way and multi-subject feedback, giving priority to the use of technical type that can provide emotional feedback and promote feedback loops; on the other hand, pay attention to the feedback process and mechanism, avoid increasing students’ dependence on computer, and strengthen learners’ subjectivity from the perspective of feedback mechanism.

There are some shortcomings in this study. First, there are few literature samples on learning satisfaction and knowledge retention in the main effect test, leading to a small number of relevant effect sizes. The independent effect size of some variables in the moderating effect test is small and unevenly distributed, which leads to the failure of the moderating effect test. Second, most of the existing empirical studies focus on the effect of feedback direction, feedback form and feedback technology type on learning performance. There are few studies involving real-time feedback, feedback frequency, learner’s prior knowledge and learner’s age, which may be important moderating variables but not included.

In the future, researchers may consider the following aspects to go deeper. First, try to explore in depth the potential boundary conditions for the effects of human-machine feedback mechanisms by using learner age, subject category, and feedback real-time as moderating variables. Second, to find more scientific and effective research methods, such as using social network analysis methods to understand human-machine interaction feedback information from more dimensions and reveal the feedback process and effect of different groups of learners. Third, to explore the effects of human-machine interaction feedback on learners’ cognitive neural activity using techniques such as electroencephalogram, near-infrared technology, and affective computing. Only by conducting cross-disciplinary research from a cross-disciplinary perspective can we fundamentally clarify the meaning and value of human-machine interaction feedback and promote the innovative development of smart learning environments.

## Data availability statement

The original contributions presented in this study are included in this article/supplementary material, further inquiries can be directed to the corresponding authors.

## Ethics statement

The studies involving humans were approved by the Ethics Committee of Pingdingshan University. The studies were conducted in accordance with the local legislation and institutional requirements. Written informed consent for participation in this study was provided by the participants’ legal guardians/next of kin.

## Author contributions

ML: Conceptualization, Data curation, Validation, Writing – original draft. KZ: Data curation, Formal analysis, Validation, Writing – original draft. GW: Investigation, Methodology, Supervision, Validation, Writing – review and editing.
